# Supplementary material for: A learning-to-rank framework for large-scale identification of unknown proteins from cryo-EM and cryo-ET maps
Source: bioRxiv. 2026 Oct 13:2026.10.12.758677. Preprint. [Version 1] doi: 10.64898/2026.10.12.758677 (PMC13715899; doi:10.64898/2026.10.12.758677)

Supplementary Information

A learning-to-rank framework for large-scale identification of unknown proteins from cryo-EM and cryo-ET maps

Qi Hu[1,2] & Georges Mer[1,2]

[1]Department of Biochemistry and Molecular Biology, Mayo Clinic, Rochester, MN, USA

[2]Structural Biology Core, Mayo Clinic, Rochester, MN, USA

Correspondence and requests for materials should be addressed to Qi Hu (hu.qi@mayo.edu) or Georges Mer (mer.georges@mayo.edu).

Supplementary Table 1 | Docking-derived features used in the final CryoAssign learning-to-rank model.

Feature	Feature type	Description	Formula
corr1	Docking score	CoLoRes unnormalized cross-correlation score	CoLoRes output
gap12	Score separation	Difference between the top two CoLoRes scores	$s_1 - s_2$
overlap_cc_strict_orig	Map-model agreement	Pearson correlation within the strict overlap region	Eq. (1), Ω = Ω_strict
overlap_cc_ local_orig	Map-model agreement	Pearson correlation within the dilated overlap region	Eq. (1), Ω = Ω_local
masked_realspace _cc_orig	Map-model agreement	Pearson correlation over the complete model mask	Eq. (1), Ω = Ω_model
map_explained _voxels	Density coverage	Number of significant map voxels explained by the candidate	|Ω_map ∩ Ω_model|
supported_ residue_fraction_z	Density coverage	Fraction of residues supported by experimental density	N_res,supported / N_res
largest_overlap_ component_ fraction_model	Structural continuity	Size of the largest connected overlap component normalized by the model-mask size	|C_max| / |Ω_model|
overlap_fragmentation _penalty	Structural continuity	Fragmentation penalty of the overlap region	1 − |C_max| / |Ω_overlap|
n_atoms_total	Candidate size	Total number of atoms in the candidate structure	N_atoms

s1 and s2 denote the highest and second-highest CoLoRes scores, respectively. Ω_strict, Ω_local and Ω_model denote the strict overlap region, the dilated overlap region and the model mask, respectively. Ω_map denotes the significant map mask, and Ω_overlap = Ω_map ∩ Ω_model denotes the overlap mask. |Ω| denotes the number of voxels in the corresponding mask. C_max denotes the largest connected component of Ω_overlap. N_res denotes the total number of residues in the candidate structure, and N_res,supported denotes the number of supported residues. A residue is considered supported if at least 50% of its sampled robust density values satisfy z ≥ z0. A threshold of z0 = 1.0 was used. N_atoms denotes the total number of atoms in the candidate structure.

Supplementary Table 2 | Performance comparison between the CoLoRes corr1 baseline and the final CryoAssign ranker on the held-out benchmark set.

	CoLoRes corr1 baseline	CryoAssign ranker
Map-wise AP	0.8420	0.9813
**MRR**	0.9464	1.0000
**Recall@P**	0.8095	0.9583
**NDCG**	0.9134	0.9940
**Top-1 hit**	0.9286	1.0000
**Top-5 hit**	1.0000	1.0000
**Top-10 hit**	1.0000	1.0000
**Mean positive rank**	12.70	2.93

Metrics were calculated for held-out map groups containing at least one positive candidate.

Recall@P denotes the fraction of positive candidates recovered within the top *P* positions, where *P* is the number of positive candidates for that map.

Top-1, Top-5, and Top-10 hit rates denote the fraction of maps for which at least one positive candidate was ranked within the top 1, 5, or 10 positions, respectively.

Mean positive rank is the mean rank of all positive candidates within each map, averaged across maps; lower values indicate better ranking performance.

MRR, mean reciprocal rank; MRR = $(1/N)\Sigma(1/r_i)$, where r_i is the rank of the first true candidate for map i.

NDCG, normalized discounted cumulative gain; AP, average precision.

Supplementary Table 3 | Cryo-EM data collection, refinement and validation statistics

	DnaK	ArnA	A2M
**Data collection and processing**			
Magnification	130,000	130,000	165,000
Voltage (kV)	300	300	200
Electron exposure (e^{-} Å^{-2})	50	50	50
Defocus range (μm)	−0.5 to −2.5	−0.5 to −2.5	−0.4 to −2.0
Pixel size (Å)	0.664	0.664	1.76
Symmetry imposed	C1	C3	D2
Initial particle images (no.)	132,536	752,726	249,772
Final particle images (no.)	41,666	150,866	68,118
Map resolution (Å)	4.2	3.9	4.5
FSC threshold	0.143	0.143	0.143
**Refinement**			
Map sharpening B factor (Å^2)	−145	−120	−160

Supplementary Figure 1 | Per-map performance of CryoAssign on the held-out benchmark set. Each page summarizes the performance of CryoAssign for one of the 14 held-out benchmark maps containing at least one manually confirmed true-positive candidate. For each map, score distributions, feature relationships, cumulative recovery curves, and per-map ranking metrics are shown. Positive candidates correspond to manually annotated true hits.

Supplementary Figure 1a

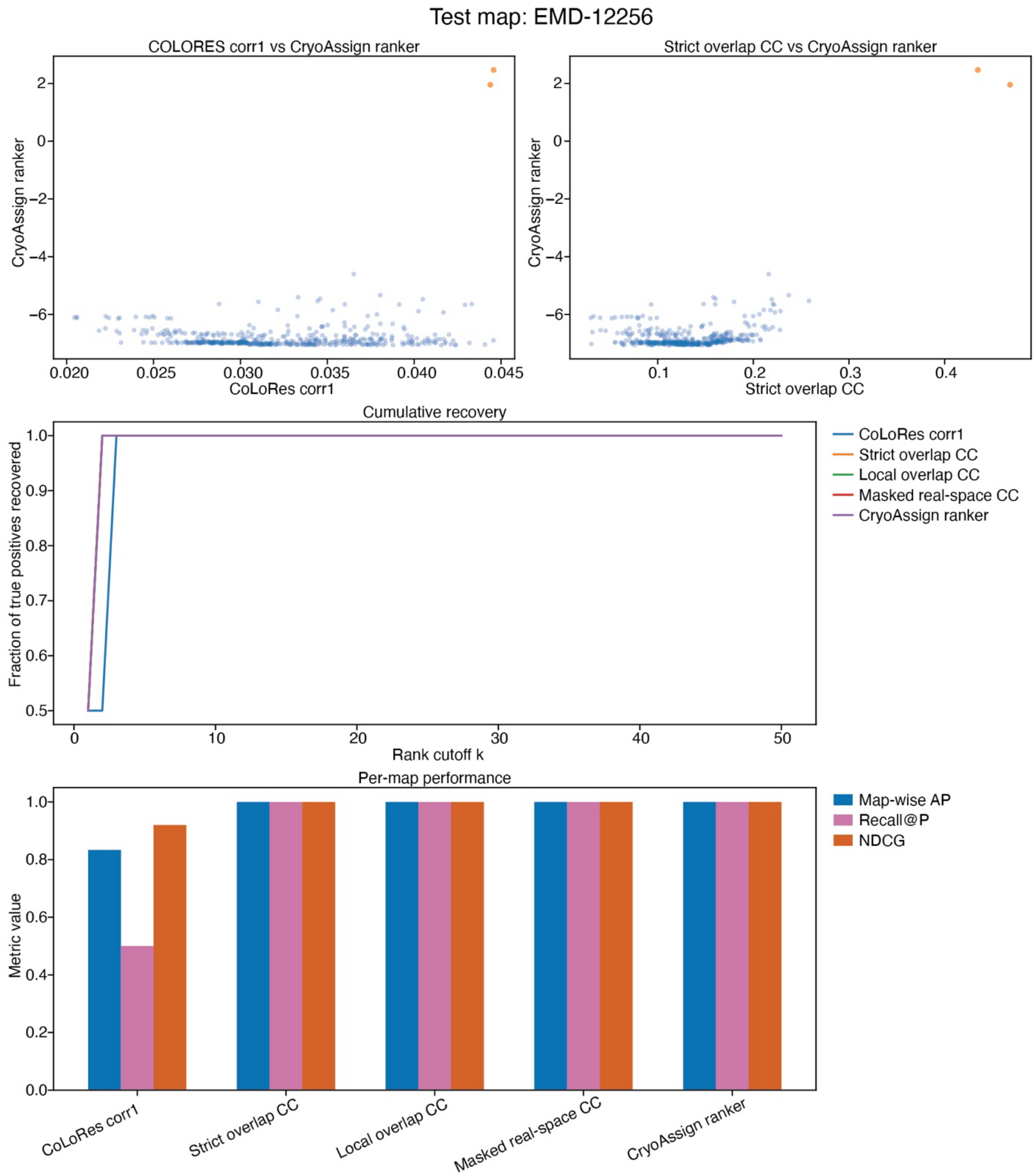

Supplementary Figure 1b

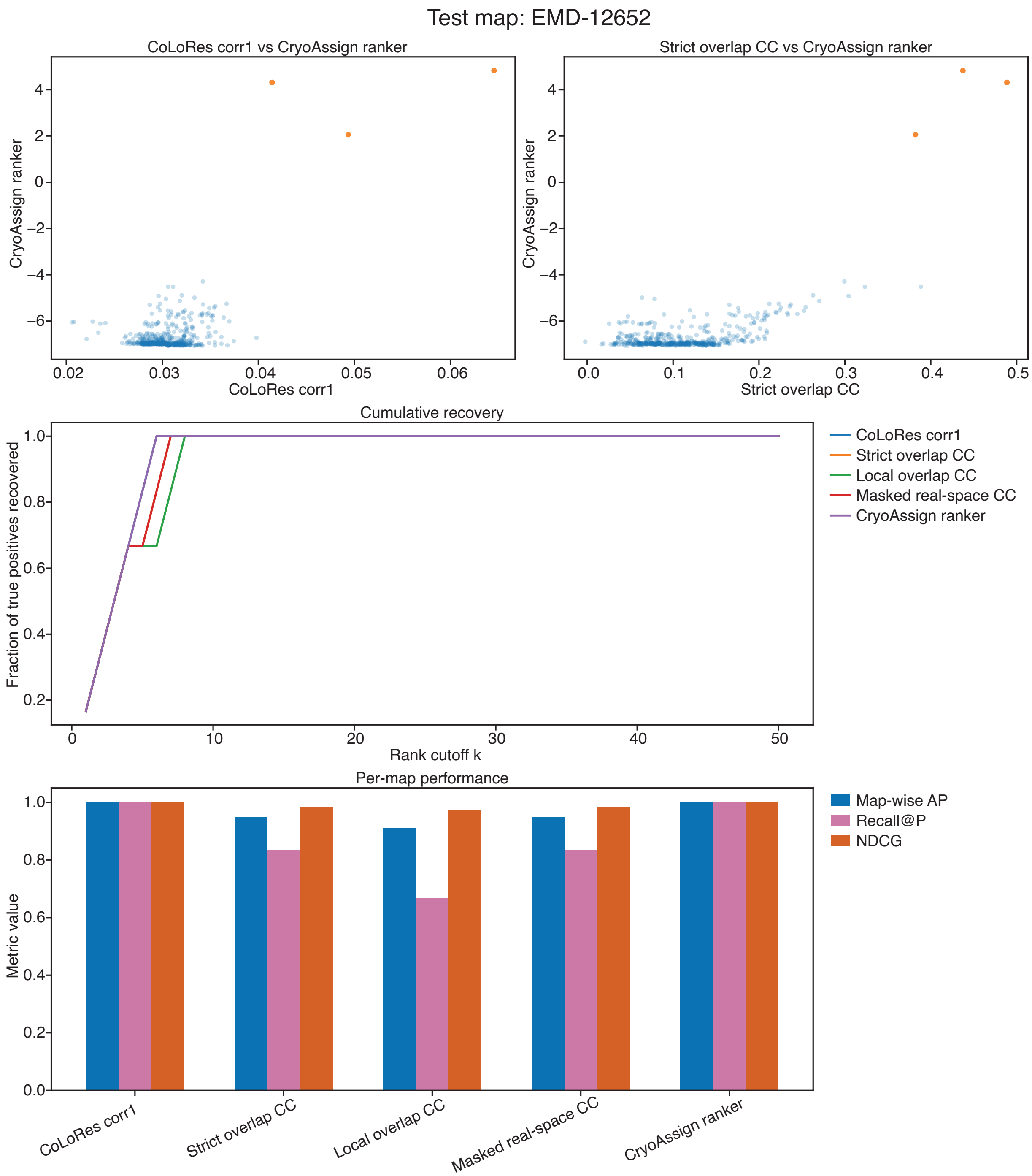

Supplementary Figure 1c

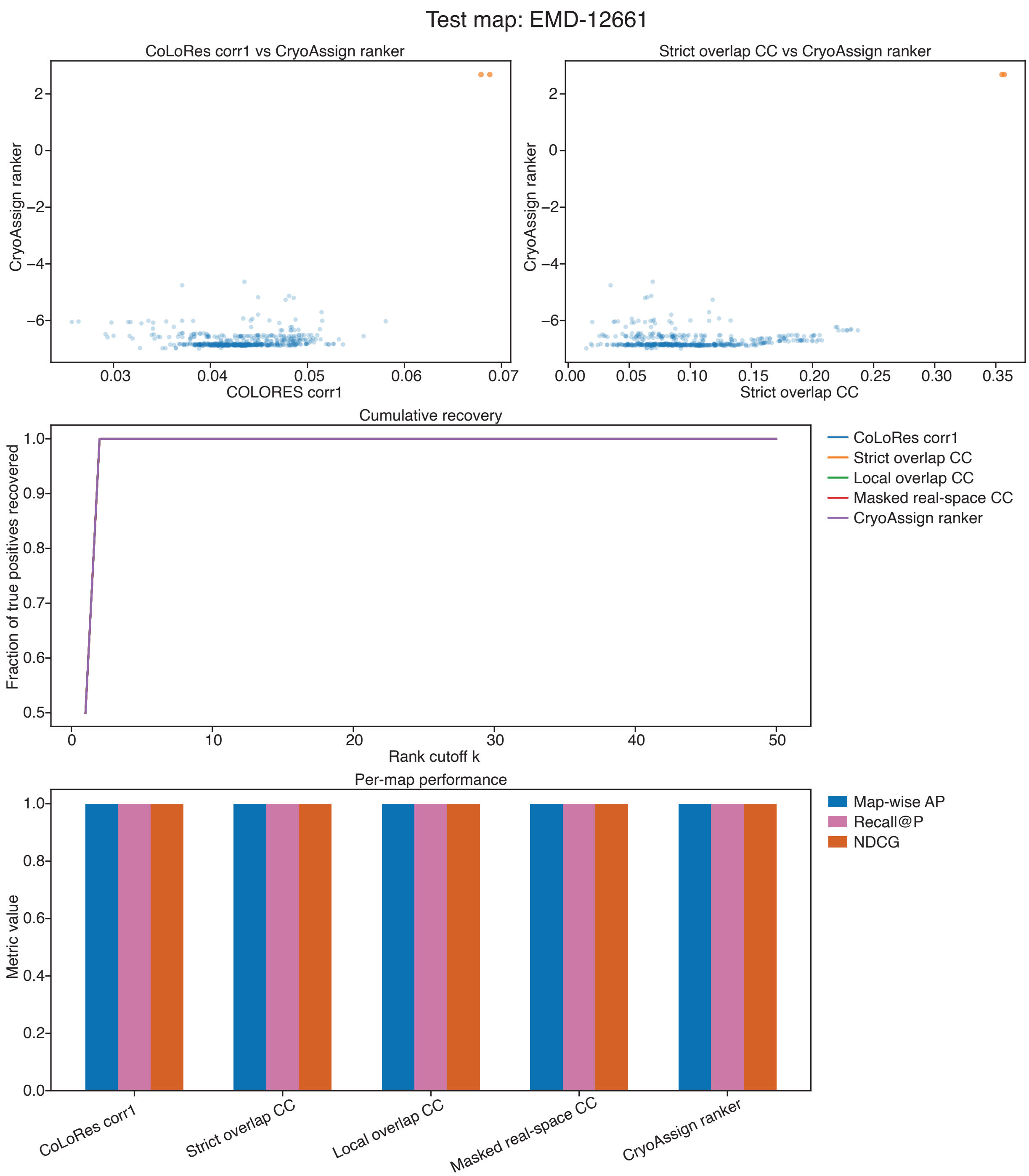

Supplementary Figure 1d

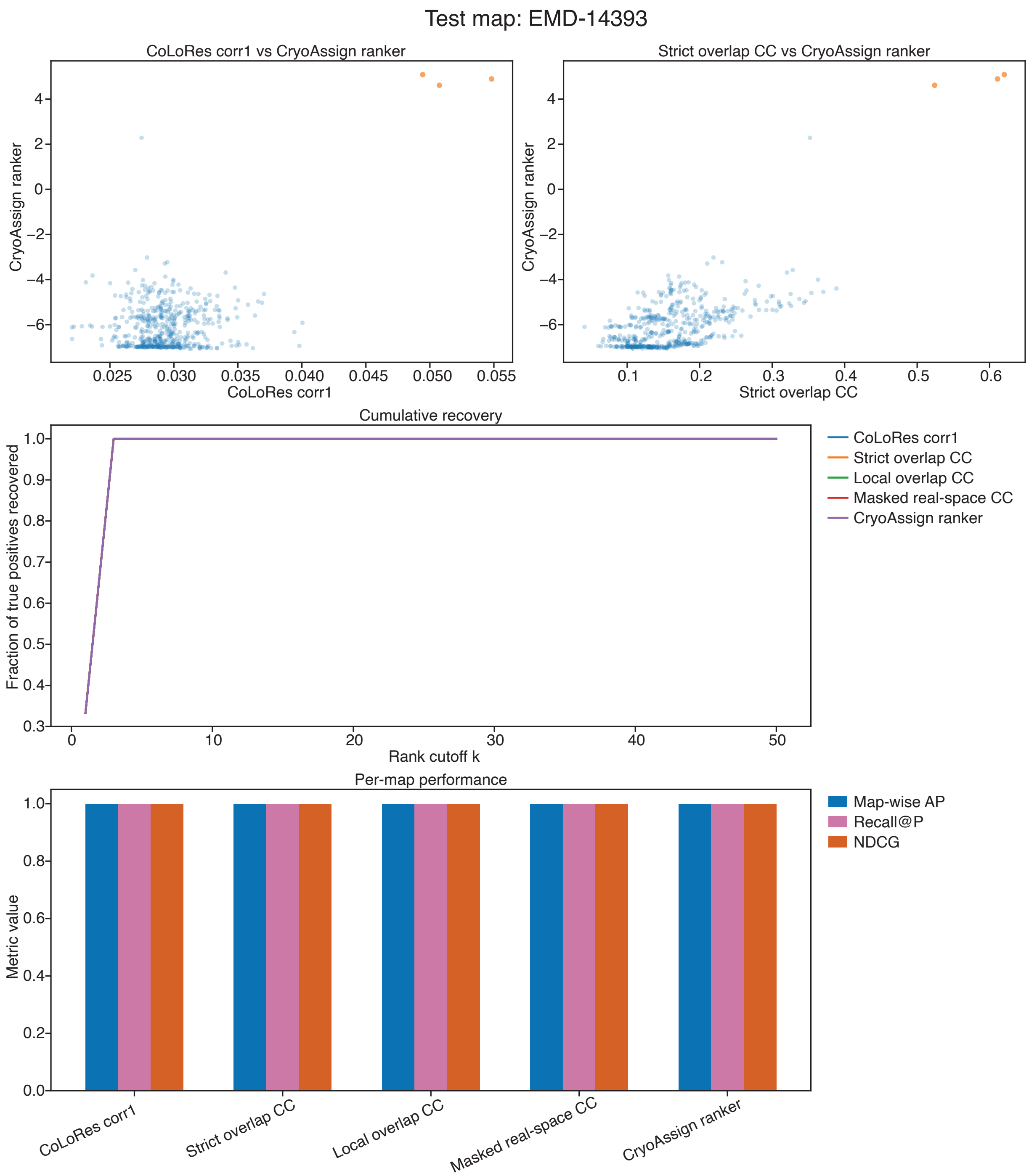

Supplementary Figure 1e

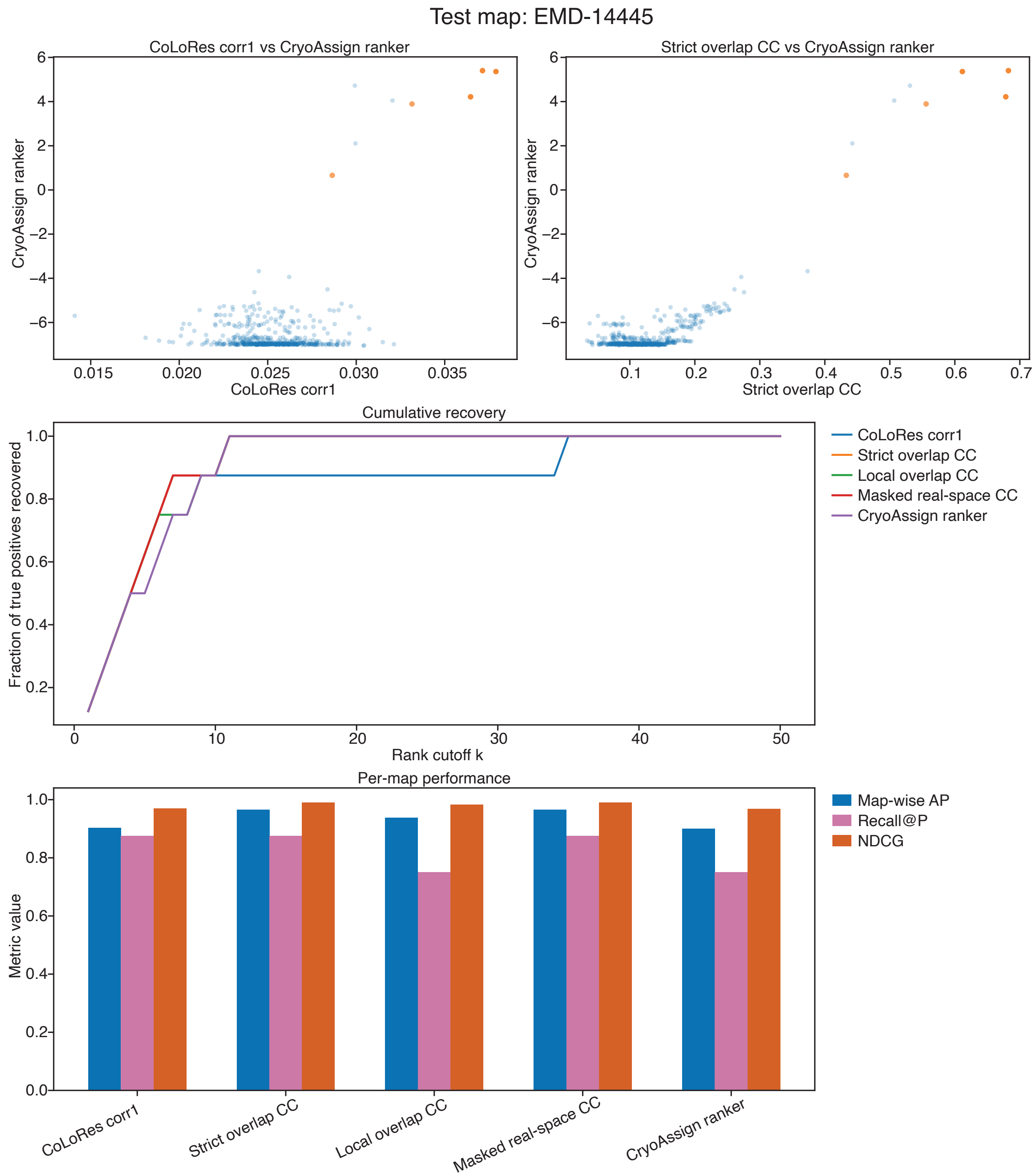

Supplementary Figure 1f

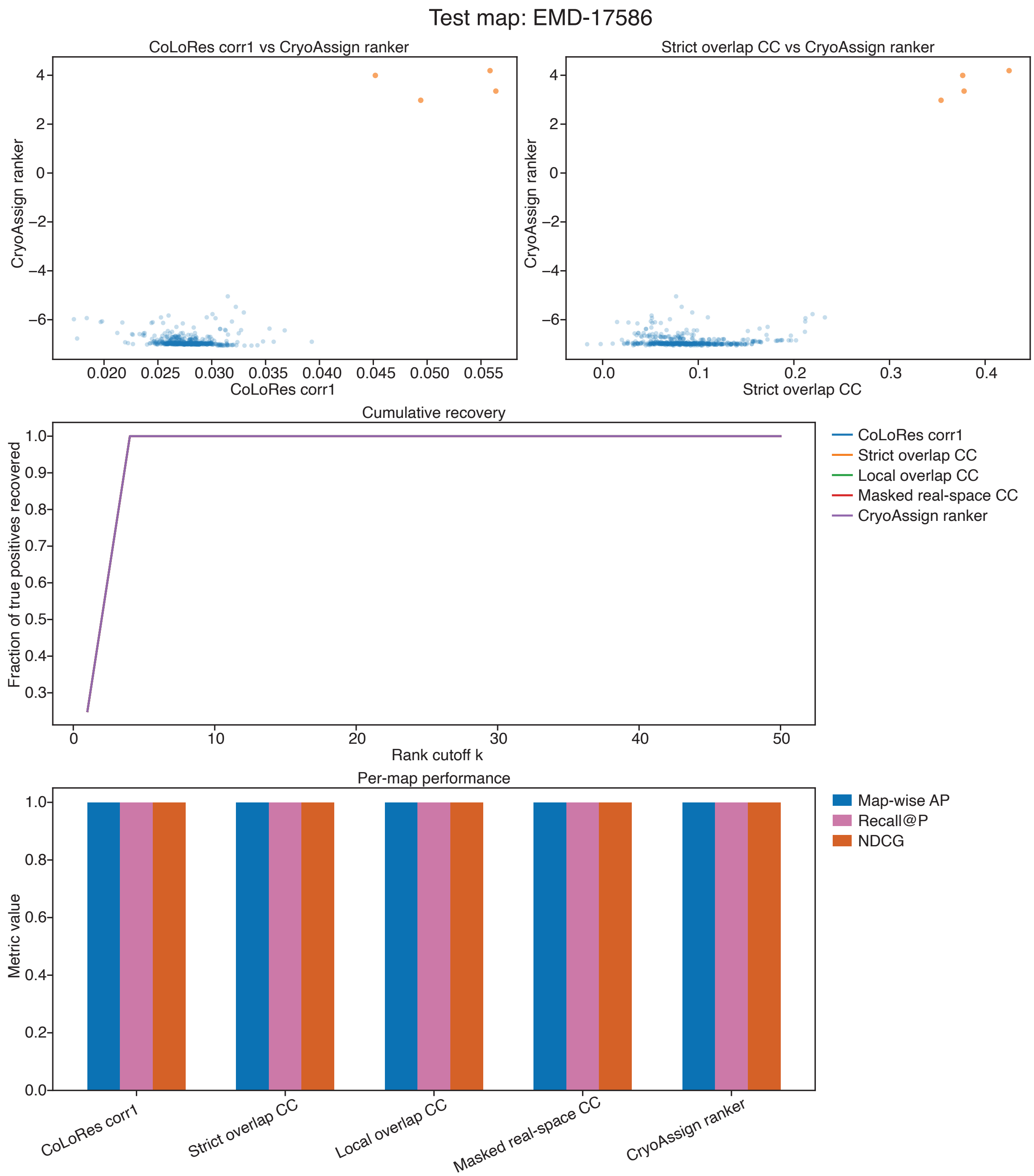

Supplementary Figure 1g

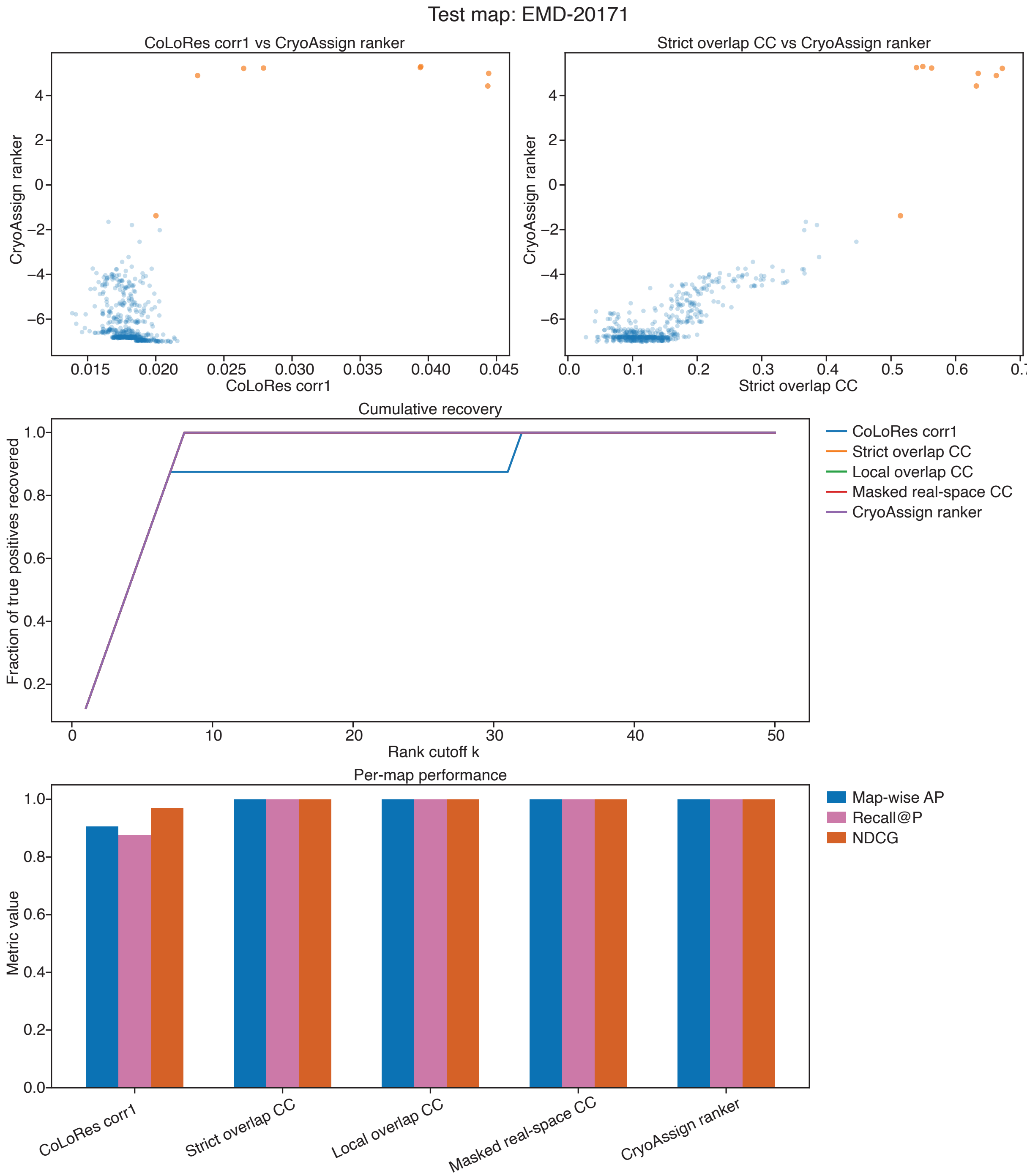

Supplementary Figure 1h

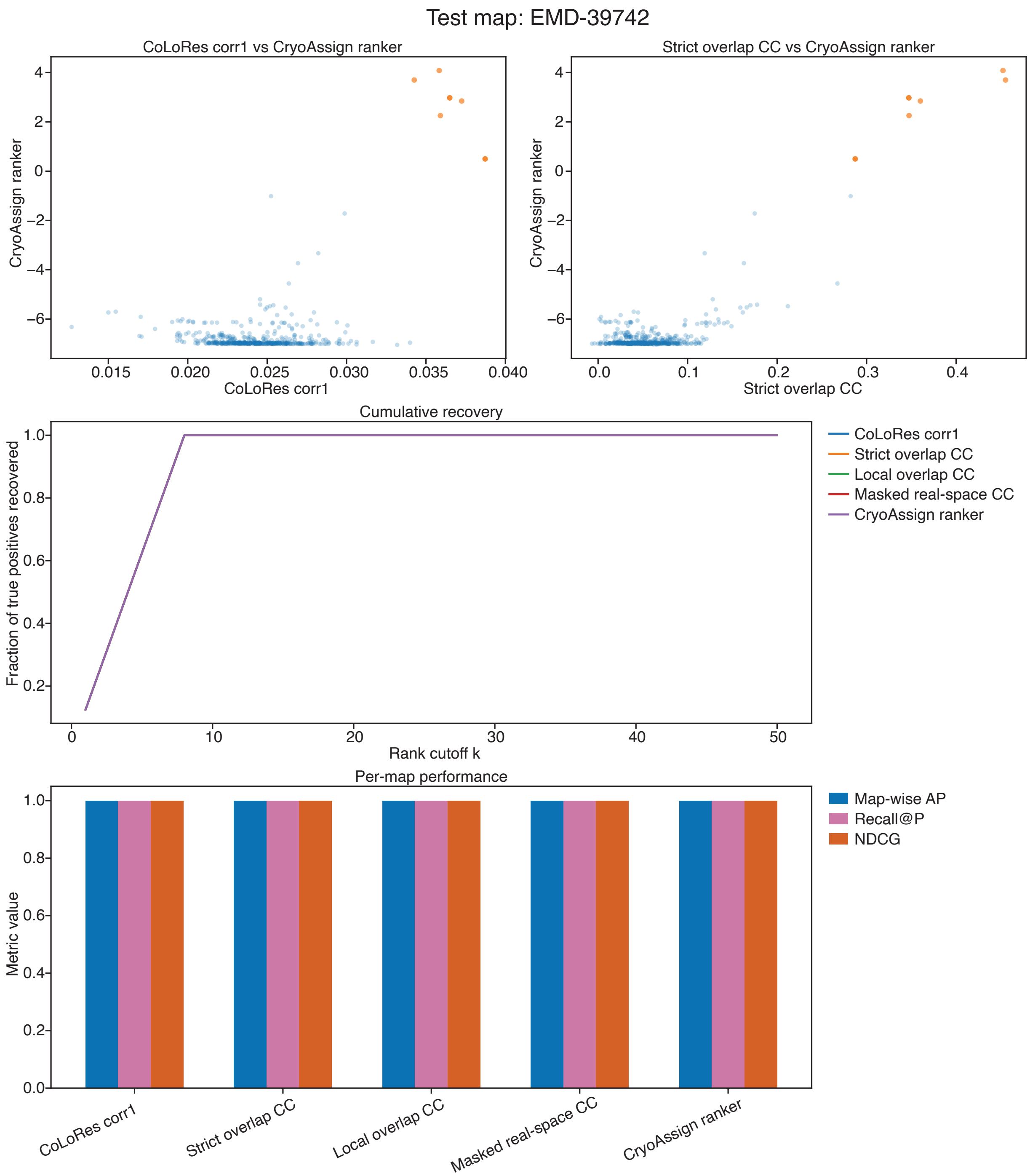

Supplementary Figure 1i

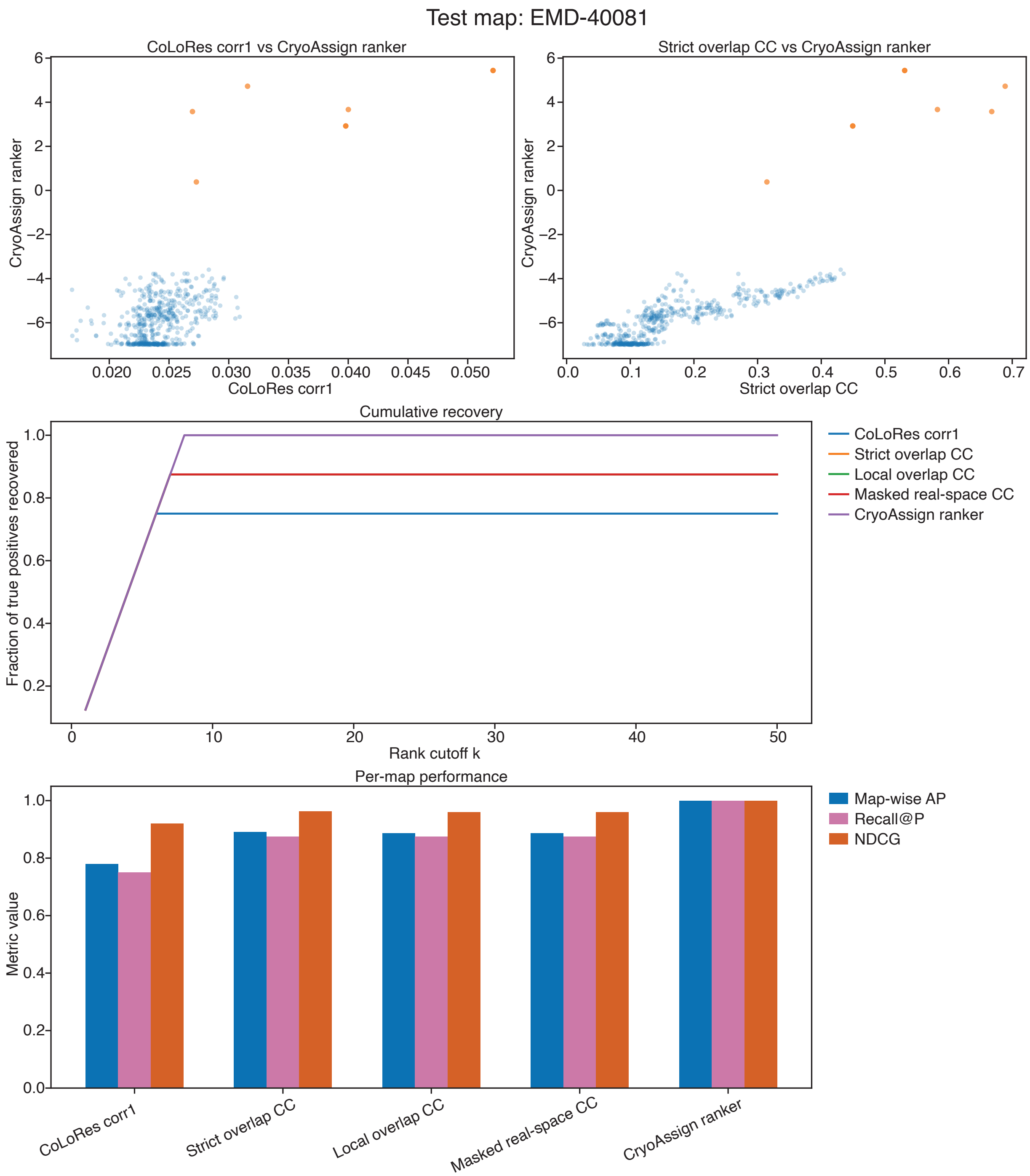

Supplementary Figure 1j

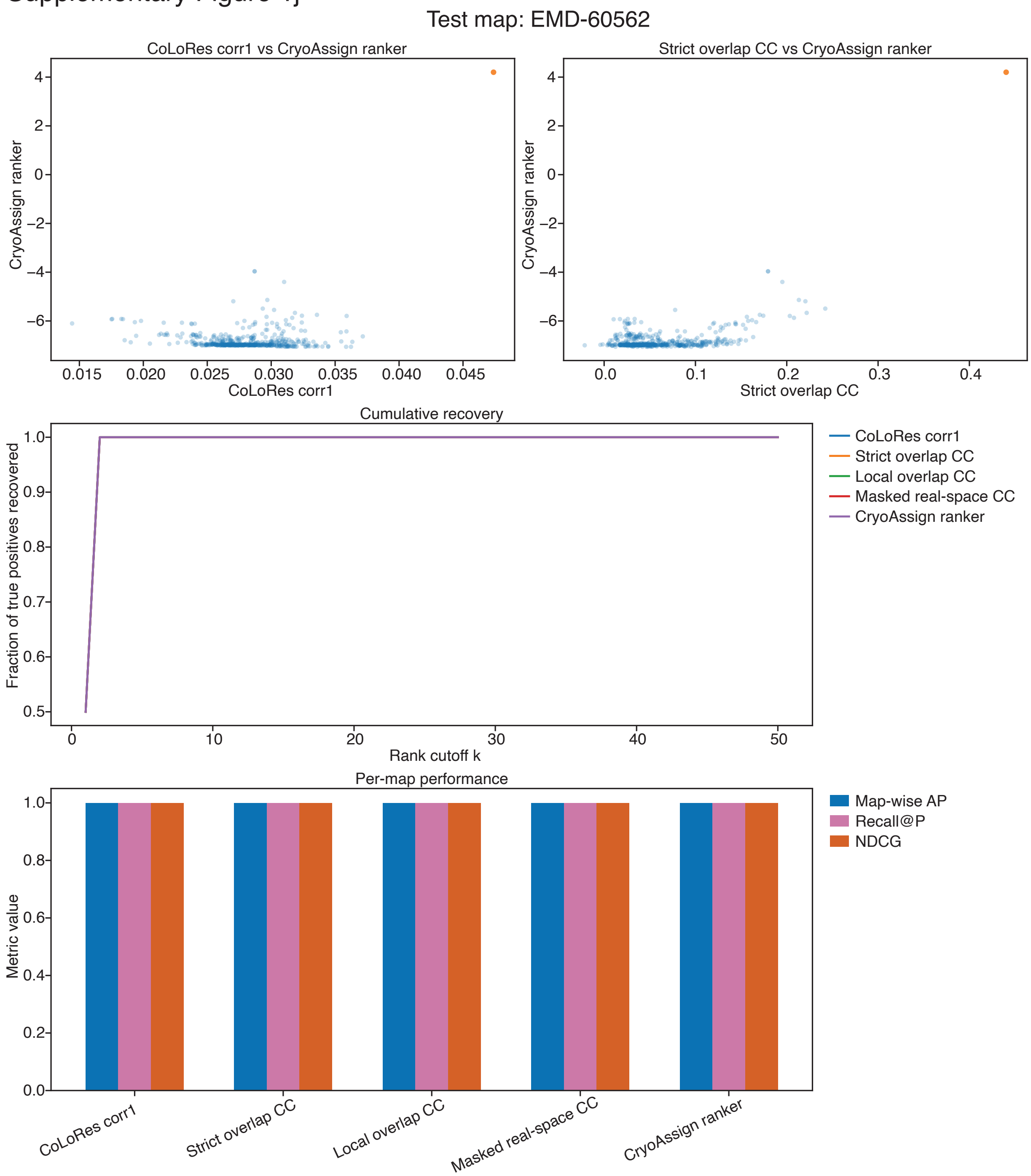

Supplementary Figure 1k

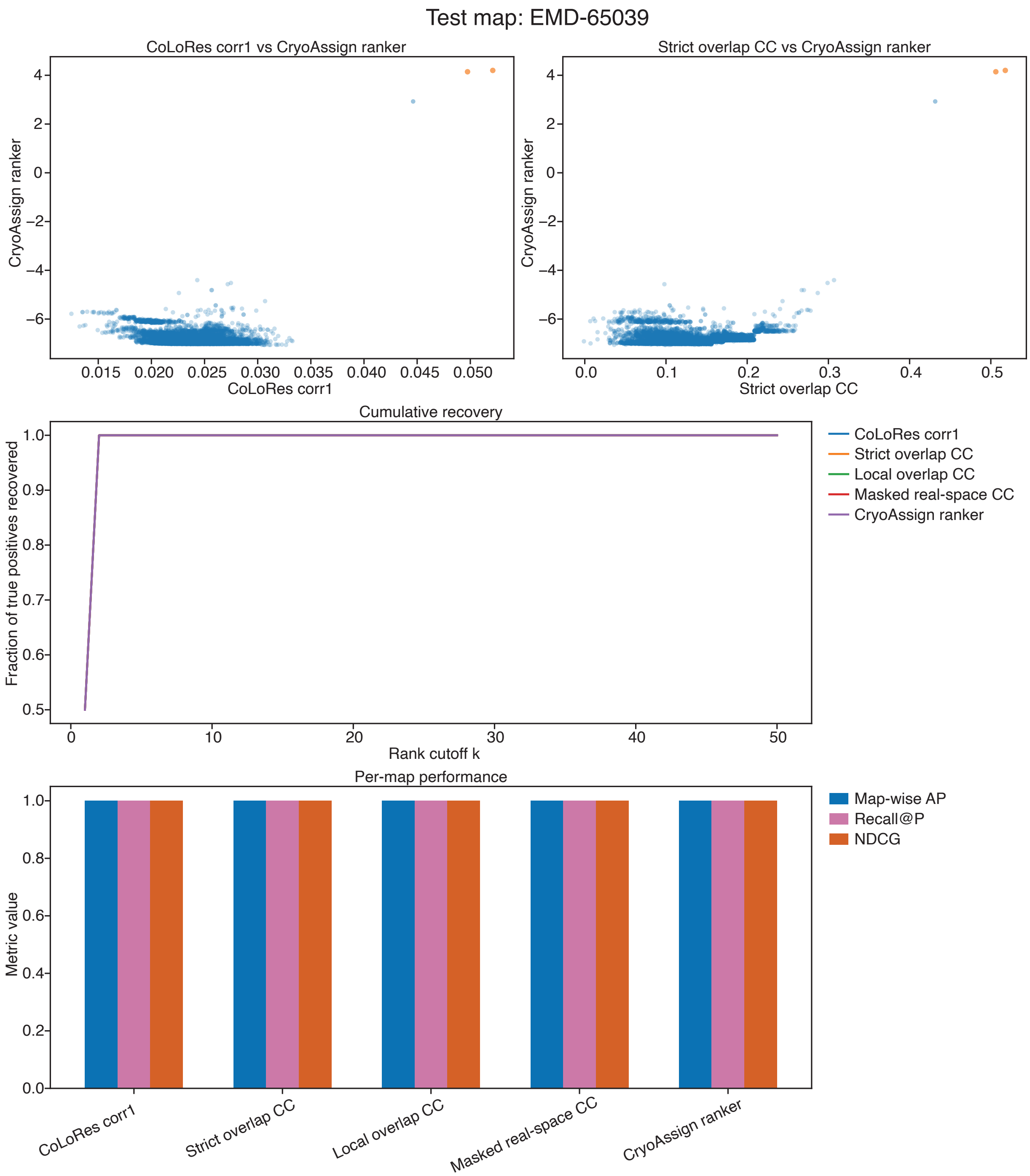

Supplementary Figure 1l

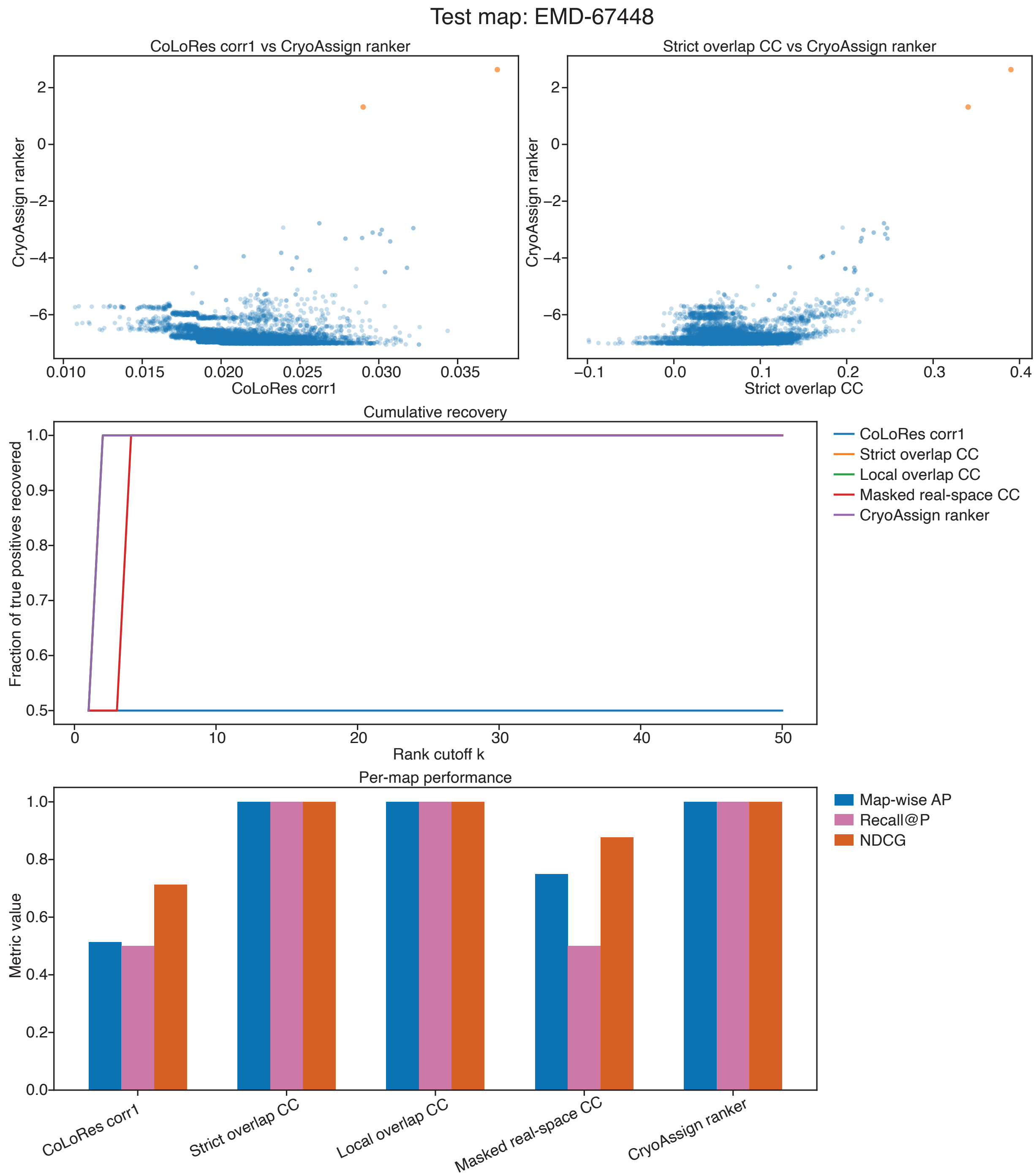

Supplementary Figure 1m

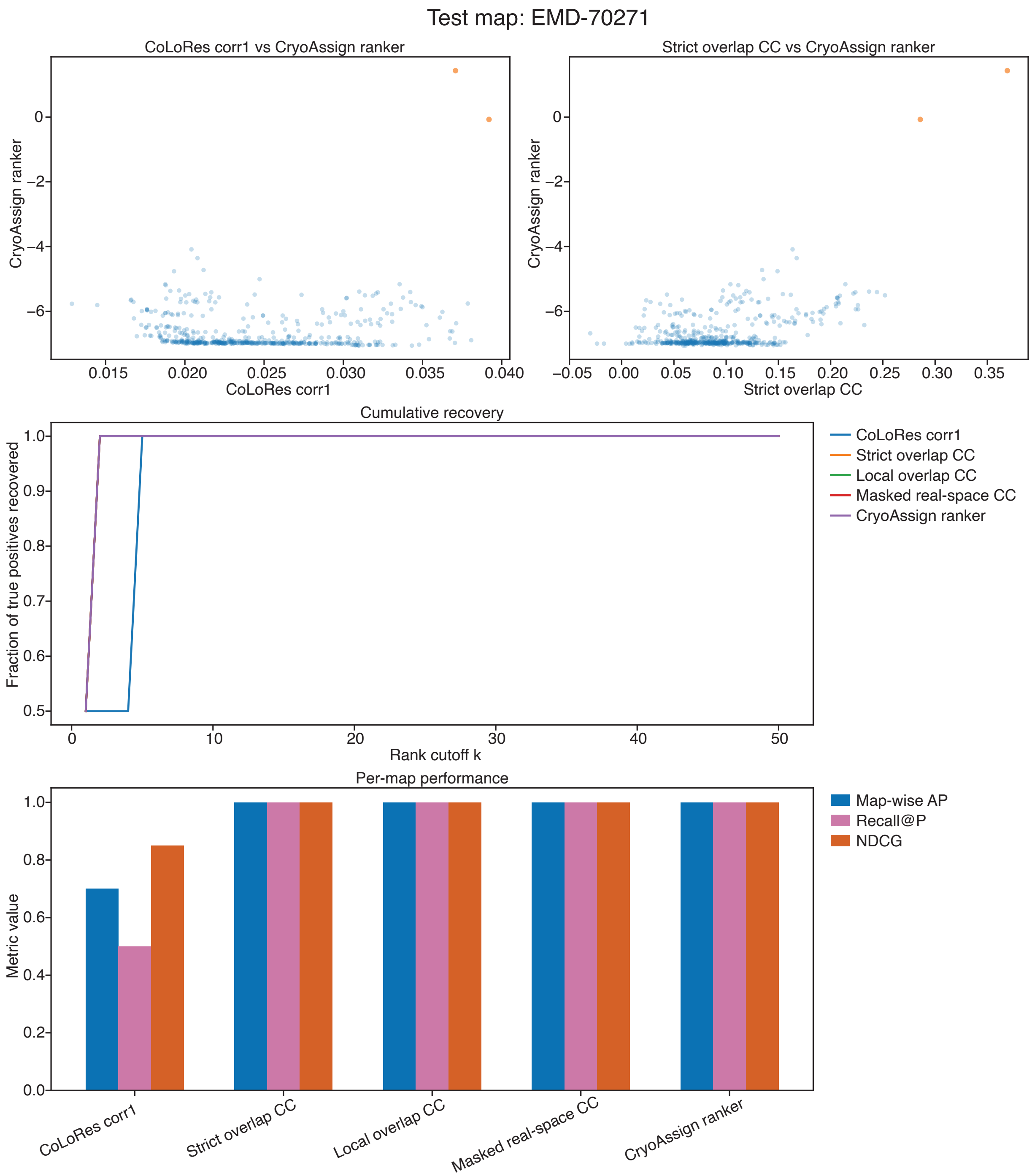

Supplementary Figure 1n

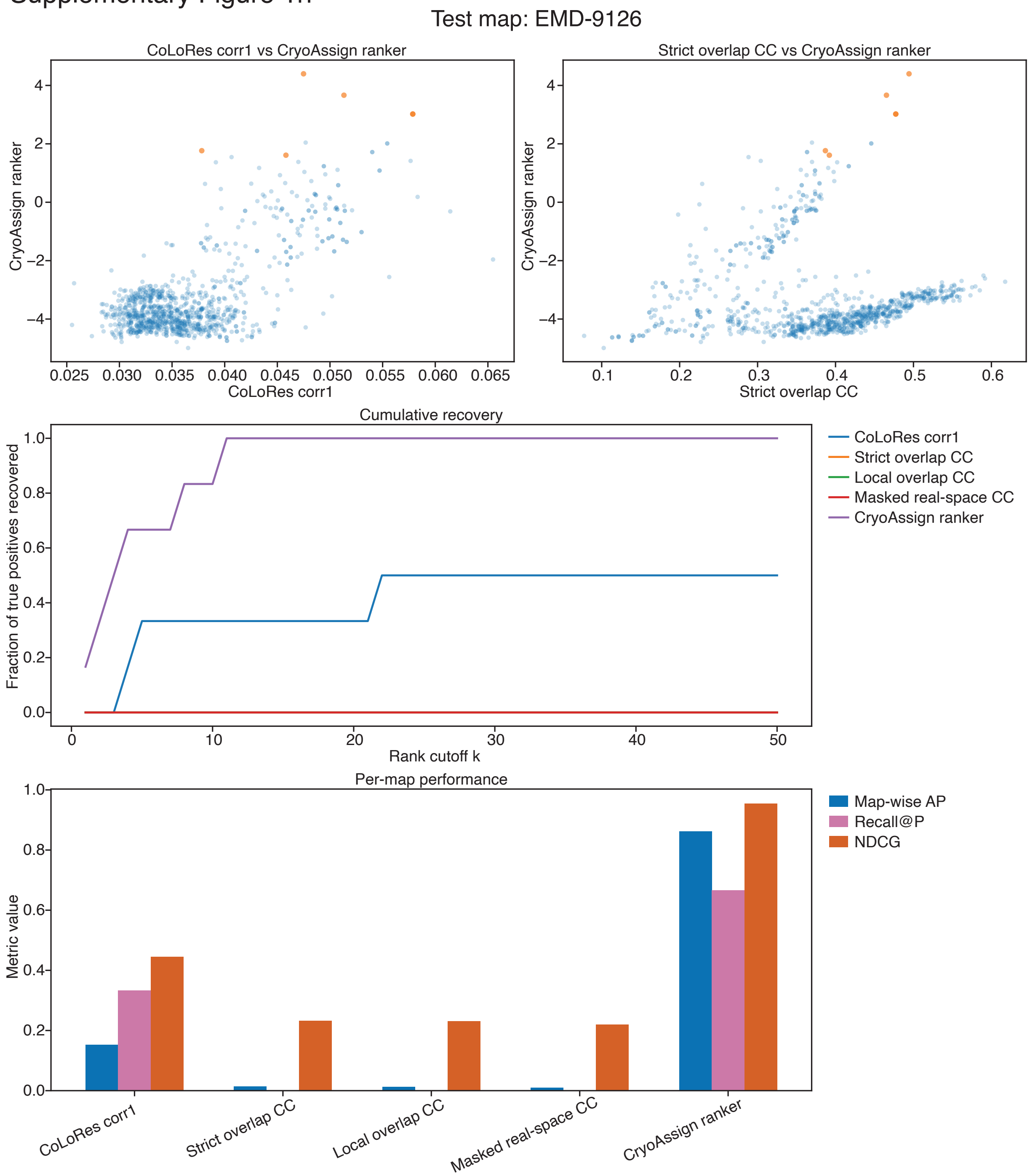

Supplementary Figure 2 | Resolution-dependent changes in CryoAssign ranker scores and CoLoRes corr1 rankings. CryoAssign was applied to EMD-9126, EMD-12661, EMD-50723, and EMD-65039 in their native forms and after progressive low-pass filtering, as described for Fig. 6. Panels follow the same map order as Fig. 6a–d. **a,c,e,g,** CryoAssign ranker scores for true-positive candidates and the 20 highest-scoring negative candidates at each filtering condition. The highest-scoring negative candidate at each condition is highlighted and connected across conditions by a dashed line. **b,d,f,h**, Ranks of the true-positive proteins obtained using the original CoLoRes corr1 score, displayed using the same grouping and axis conventions as in Fig. 6a–d.

Supplementary Figure 2

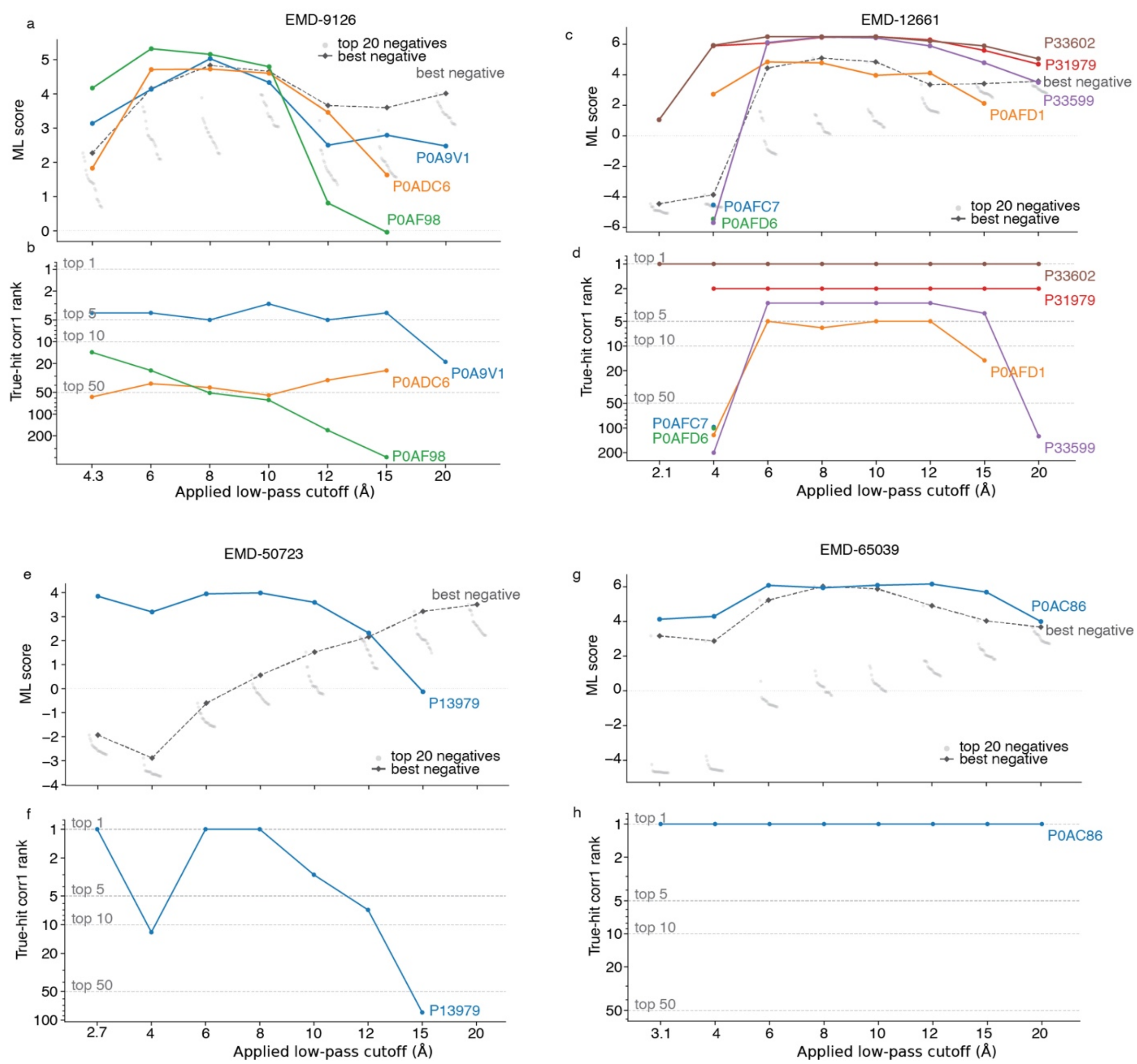

Supplement: Supplementary file 1 [file media-1.pdf]
